# Methotrexate-Induced Pneumonitis in a Patient with Chronic Chikungunya Arthritis

**DOI:** 10.1590/0037-8682-0136-2025

**Published:** 2025-07-07

**Authors:** Jobson Lopes de Oliveira, Igor Albuquerque Nogueira, Afonso Rocha Eisele, Luis Arthur Brasil Gadelha Farias

**Affiliations:** 1 Centro Universitário Christus, Faculdade de Medicina, Fortaleza, CE, Brasil.; 2 Universidade Federal do Ceará, Faculdade de Medicina, Departamento de Medicina Clínica, Fortaleza, CE, Brasil.; 3 Universidade de São Paulo, Hospital das Clínicas da Faculdade de Medicina de Ribeirão Preto, Ribeirão Preto, SP, Brasil.; 4 Universidade de São Paulo, Departamento de Doenças Infecciosas do Hospital das Clínicas, Laboratório de Investigação Médica - LIM 49, São Paulo, SP, Brasil.; 5 Hospital São José de Doenças Infecciosas, Fortaleza, CE, Brasil.

Methotrexate (MTX) is a cornerstone in the treatment of inflammatory arthritis owing to its immunomodulatory effects. Its use has expanded to the management of post-viral inflammatory conditions, including chronic arthritis caused by Chikungunya virus (CHIKV) infection, which may mimic rheumatoid arthritis (RA)^1^. Although effective, MTX poses a risk of adverse effects, including rare but potentially life-threatening complications such as pneumonitis^2^ ^,^ ^3^.

We present the case of a 50-year-old Brazilian woman who developed MTX-induced pneumonitis during treatment for post-CHIKV arthritis. Initial presentation included acute-onset fever, rash, and polyarthritis. Serological testing confirmed a recent CHIKV infection (IgM-positive), while investigations for other causes of inflammatory arthritis, including RA and systemic lupus erythematosus, were negative. Symptoms improved with corticosteroids, and MTX (15 mg/week) was initiated as a steroid-sparing agent.

**FIGURE 1:**
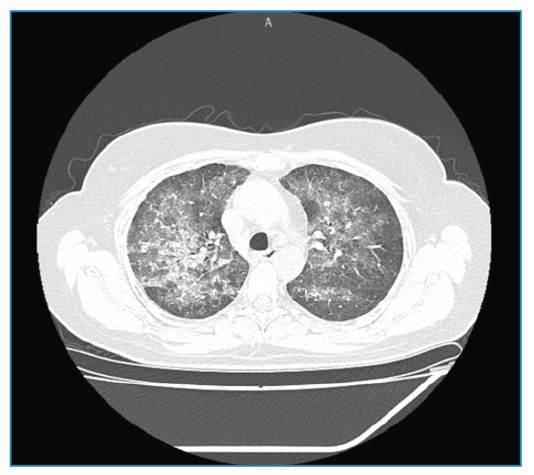
High-resolution computed tomography scan of the chest showing diffuse bilateral ground-glass opacities with interlobular septal thickening that predominantly affected the upper lobes and peribronchovascular regions. These radiological features are characteristic but not pathognomonic of MTX-induced pneumonitis.

Three weeks later, the patient developed progressive dyspnea, dry cough, and intermittent fever. Infectious etiologies, such as SARS-CoV-2 and HIV, were excluded, and high-resolution computed tomography (CT) revealed diffuse bilateral ground-glass opacities and interlobular septal thickening. These findings were predominantly observed in the upper lobes and peribronchovascular regions, consistent with hypersensitivity pneumonitis (Figure 1). MTX was discontinued. Methylprednisolone pulses was applied, resulting in rapid clinical improvement without symptom recurrence, thus supporting the diagnosis of MTX-induced pneumonitis.

MTX-induced pneumonitis typically manifests as an acute or subacute hypersensitivity reaction, which often occurs within the first year of therapy^3^. Radiological findings may resemble those of nonspecific interstitial pneumonia, with histopathology revealing lymphocytic infiltrates and non-caseating granulomas.

Given the efficacy of MTX in treating post-CHIKV arthritis, clinicians should be vigilant of pulmonary toxicity, particularly during the early stages of treatment. Risk factors include old age, pre-existing lung disease, diabetes mellitus, and hypoalbuminemia^2^ ^,^ ^3^. Early recognition and prompt withdrawal of MTX are crucial for recovery and the prevention of disease progression.
